# Breakthrough Cancer Pain Is Associated with Spinal Gap Junction Activation via Regulation of Connexin 43 in a Mouse Model

**DOI:** 10.3389/fncel.2017.00207

**Published:** 2017-07-17

**Authors:** Xin Li, Siqing Jiang, Hui Yang, Qian Liao, Shousong Cao, Xuebin Yan, Dong Huang

**Affiliations:** ^1^Department of Pain, The Third Xiangya Hospital and Institute of Pain Medicine, Central South University Changsha, China; ^2^Department of Pharmacology, School of Pharmacy, Southwest Medical University Luzhou, China

**Keywords:** breakthrough cancer pain, spinal cord, gap junction, connexin 43, phosphorylation, Gap26

## Abstract

Breakthrough cancer pain (BTcP) is a high-intensity, short-duration, unpredictable and uncontrollable pain. Recent studies have shown that activation of gap junction (GJ) in spinal cord plays an important role in the pathogenesis of BTcP. We examined the expressions of Glial fibrillary acidic protein (GFAP), connexin (Cx) 43 protein and phosphorylation of Cx43 (p-Cx43) in the spinal cord of mice. In addition, we investigated the effects of Gap26, a selective GJ blocker, on the expressions of GFAP, Cx43 and p-Cx43 in BTcP mice. We found that the expressions of GFAP and Cx43 proteins were significantly upregulated while p-Cx43 was down-regulated in the spinal cord in a mouse model of BTcP. The overexpression of Cx43 protein in the spinal cord increased GJ formation and enhanced BTcP. The variation of the ratio of p-Cx43/T-Cx43 (total Cx43) affected the function of GJ to induce BTcP. Furthermore, BTcP was alleviated by Gap26 via reducing pain hypersensitivity. The inhibition of Cx43 and p-Cx43 by Gap26 attenuated BTcP but the p/T ratio of Cx43 remained unchanged in BTcP mice. We reveal that the expression and phosphorylation of Cx43 affected BTcP and GJ activation facilitated BTcP via a Cx43-mediated signaling in the spinal cord. The finding may provide a scientific rationale for discovery and development of novel therapeutic targets for the treatment of BTcP clinically.

## Introduction

Cancer is one of the most common diseases and the leading causes of morbidity and mortality worldwide, with 14.1 million new cancer cases and 8.2 million deaths occurred in 2012 (Torre et al., 2015). Cancer-related pain is experienced in 70%–90% of the patients with advanced cancer and adversely affected their productivity and quality of life significantly (Schrijvers, 2007). Cancer-related pain is broadly divided into two types of persistent background pain and breakthrough cancer pain (BTcP). BTcP has the characters of a temporal variability in pain intensity with peaks and interrupting the effect of adequate analgesia (Porta-Sales et al., 2010). The clinical manifestations of BTcP are rapid onset within 3 min, severe intensity with the numerical rating scale (NRS) more than 7, short duration with less than 30 min, and multiple daily frequency with more than four episodes per day (Zeppetella and Ribeiro, 2003). Because of its unpredictable and uncontrollable, BTcP is difficult to be managed clinically so it has become an emerging field of research recently. As a consequence, a better knowledge to understand its pathogenesis and pathophysiology is required in order to identify the etiology and new therapeutic targets of BTcP for timely relieving and even preventing its occurrence. We have previously established a reliable animal model of BTcP induced by endothelin-1 (ET-1), which can mimic the clinical manifestations and neurophysiologic changes in cancer patients with BTcP (Tang et al., 2016). It provides a useful platform for further research of the pathogenesis and pathophysiology of BTcP and related intervention.

Currently, most studies of BTcP are based on the clinical characteristics and drug treatment (Mercadante et al., 2016), but the mechanisms still remain unclear. It is generally believed that central and peripheral sensitizations play a role, at least in part, in the pathogenesis and pathophysiology of BTcP (Svendsen et al., 2005), which may provide a new way for a promising area of pharmacological interventions. Recent studies have shown that activation of astrocytes in spinal dorsal horn plays an important role in sensitization of pain presented as hyperalgesia and persistent pain (Svensson and Brodin, 2010). The functional alterations of astrocytes in the dorsal horn of the spinal cord can ultimately affect the excitability of neurons, therefore, promoting the generation and conduction of pain (Sasaki et al., 2014). Information networks can be built between astrocyte-astrocyte and/or astrocyte-neuron through a variety of forms (Ji et al., 2013). Studies have implicated gap junctions (GJs) of the spinal cord as a new connection form in the pathogenesis of some pain with similar manifestations as that of BTcP (Jeon and Youn, 2015). Connexin (Cx) 43 is a basic functional protein of the GJ channel and is important for cell-to-cell electrical coupling. Expression of Cx43 affects the formation of GJ (Alvarez-Maubecin et al., 2000) and regulation of calcium oscillation in astrocytes (McClain et al., 2014), then regulates the signaling pathway between astrocytes or astrocyte and neuron in the central nervous system.

The activation state of astrocytes and the expression or functional changes of Cx43 may play an important role in the pathogenesis of BTcP. In addition, phosphorylation or dephosphorylation of Cx43 has a very important impact on the GJ channel function (Laird, 2005). Under the stimulation or certain pathological conditions, Cx43 can be catalyzed by phospholipase as dephosphorylated form to up-regulate the function of GJ. Protein kinase (PK) subtypes and their regulatory proteins may be different in various cells with different effects on the function of GJ. Studies have found that protein kinase A (PKA) can lead to phosphorylation of Cx43 (p-Cx43) in Ser368 site, thereby affecting the Cx43 conformation and down-regulating the function of GJ channel (TenBroek et al., 2001). Protein kinase C (PKC) can also phosphorylate Ser368, Ser372 and Ser262 sites of Cx43, the phosphorylation of Ser368 affects protein conformation of C-terminal peptide chain and down-regulates the function of GJ *in vitro* (Bao et al., 2004; Ek-Vitorin et al., 2006). As a consequence, abnormal regulation or regulatory alteration of phosphorylation states of Cx43 may be the structural basis for the pathology of BTcP.

Here, we used an established mouse model of BTcP induced by ET-1 to investigate the regulation of Cx43 in the spinal cord as a structural basis for BTcP. We also studied the regulation of abnormal signal transduction in the spinal cord and the associated molecular mechanisms related to BTcP.

## Materials and Methods

### Reagents

Morphine hydrochloride injection solution (10 mg/ml) was purchased from Northeast Pharmaceutical Group Shenyang First Pharmaceutical Co. LTD, (Shenyang, Liaoning, China) and diluted with normal saline (NS, 0.9% sodium chloride) to a concentration of 0.5 mg/ml. ET-1 was purchased from Sigma-Aldrich (St. Louis, MO, USA) and dissolved in distilled deionized water (DD water) at a concentration of 75 μg/ml (30 pmol/μl). GJ blocker peptide Gap26 was purchased from ApexBio technology (Houston, TX, USA), dissolved at a concentration of 10 mg/ml in sterile distilled DD water based on a previous report showing its ability to modulate GJ channels (Desplantez et al., 2012). Dubecco’s modified Eagle’s medium (DMEM) with high glucose medium, D-Hank’s solution and fetal bovine serum (FBS) albumin were purchased from Gibco Company (Gaithersburg, MD, USA).

### Animals

Five to six week-old specific-pathogen-free (SPF) grade male C57BL/6 mice (body weight 18–20 g) were purchased from the Experimental Animal Center of Central South University (Changsha, Hunan, China). The mice were housed up to 5 per cage under standard conditions (12 h light/dark cycle, 22 ± 1°C controlled ambient temperature, 50%–70% relative humidity) with *libitum* free access to food and water. A total of 135 mice with 5–15 mice in each group were used in this study. The experimental procedures were approved by the Committee on Animal Care and Use of The Third Xiangya Hospital of Central South University (Changsha, Hunan, China) with an approved protocol (LLSC(LA) 20213-012) and were consistent with the ethical guidelines recommended by the International Association for the Study of Pain in conscious animals (Zimmermann, 1983). Efforts have been made to minimize animal suffering.

### Cell Culture

Lewis lung cancer (LLC) cells (a murine lung adenocarcinoma cell line) were obtained from cell center of XiangYa Central Experiment Laboratory in Central South University (Changsha, Hunan, China). The cells were cultured with DMEM (high glucose) supplemented with 10% FBS in a humidified incubator with 5% CO_2_ at 37°C and grown to ~80% confluence in 75 cm^2^ flasks and the medium was changed every 2 days as described previously (Huang et al., 2008). Then, the cells were trypsinized to make cell suspensions and counted automatically by a TC10^TM^ automated cell counter (Bio-Rad, Hercules, CA, USA). The cells were centrifuged at 600× *g* and resuspended at the final concentration of 2 × 10^5^/μl in D-Hank’s solution prior to implantation into mice.

### Establishment of a Mouse Model of BTcP

Mice were anesthetized with intraperitoneal (i.p.) of 2% pentobarbital sodium at the dose of 50 mg/kg, then 10 μl (2 × 10^5^/μl) of LLC cell suspension was injected into the femur of left hind limb with a microinjector to establish a metastatic bone cancer pain (BCP) model as described previously (Huang et al., 2008). At post-implantation day (PID) 16, 20 μl of ET-1 (18 μg/kg, 150 pmol) was injected with a microsyringe into the femoral mass (tumor) of the mice 30 min after morphine treatment (6 mg/kg, i.p.) daily for 3 days, same volume of sterile DD water was used for vehicle control.

### Experimental Design and Drug Administration

The experiments were performed for pain behavioral tests of the paw withdrawal mechanical threshold (PWMT) and hind limb use score to evaluate pain threshold in male C57BL/6 mice and associated mechanistic studies. First, 75 mice were randomly divided into five groups: (1) control group: mice have no treatment; (2) sham group: mice were injected with D-Hank’s solution instead of LLC cells into the femur bone of left hind limb; (3) BCP group: mice were injected with LLC cells into the femur bone of left hind limb as described above (Huang et al., 2008); (4) BCP-morphine group: BCP mice were treated i.p. with morphine at 6 mg/kg/dose and injected DD water instead of ET-1 into the tumors, daily for 3 days; and (5) BTcP group: BCP mice were injected 18 μg/kg (150 pmol) ET-1 into the tumors of mice 30 min after morphine treatment, daily for 3 days. Then, the pain behaviors, proteins expressions of Glial fibrillary acidic protein (GFAP), Cx43 and p-Cx43 in lumbar spinal cords were studied. In addition, 60 mice were randomly divided into four groups for pain behaviors assessment to evaluate the effect of Gap26: (1) control, normal mice were treated with Gap26; (2) BCP mice were treated with Gap26; (3) BCP-morphine mice were treated with Gap26; and (4) BTcP mice were treated with Gap26. Gap26 was administered at 5 mg/kg/day by intrathecal injection using a microinjector (10 μl of volume) 30 min before morphine injection, daily for 3 days. At the end of experiment, the lumbar spinal cords of mice were removed for analysis of the expression levels of GFAP, Cx43 and p-Cx43 proteins.

### Pain Behavioral Tests

The pain behavioral tests of PWMT and hind limb use score were carried out at the baseline (pretreatment), PIDs 7, 13 and 15–18 at about the same time in the morning to avoid different influences on mice from the variation of circadian rhythm and other activities.

PWMT was performed as previously described (Quang and Schmidt, 2010). The mice were placed in the plastic cages and their paws accessing to a wire grid floor for 30 min before testing. The mid-plantar of mice left hind paws were connected with the rigid tips of electric Von Frey anesthesiometer (IITC Life Science Inc, Woodland Hills, CA, USA). The mice would lift the foot when the tip pressure was slowly increased for several seconds, and the pain threshold was recorded automatically for withdrawal responses. The tests were performed three times for each mouse with a test interval of 3 min. The results were calculated from the average of three tests.

Hind limb use score was performed as previously described (El Mouedden and Meert, 2005). Briefly, the mice were placed in the plexiglas cages with a souvenir plate (50 cm × 20 cm × 30 cm) for the score assessment of spontaneous pain after the mice walked 10 min for acclimation. The hind limb use score was graded as 0 = no use of left hind paw, 1 = little use of left hind paw, 2 = occasional use of left hind paw, 3 = lameness and 4 = normal use of left hind paw.

### Western Blotting

Mice were weighed and anesthetized with pentobarbital (150 mg/kg) by i.p. injection at 1 h after ET-1 treatment in PIDs 16–18. Then, the lumbar spinal cords of mice were taken and the tissues were homogenized in a lysis buffer containing phosphatase and protease inhibitor (Sigma-Aldrich, St. Louis, MO, USA). After an electrophoretic separation on 12% SDS–PAGE gel, the protein samples were transferred to a membrane to form blots. The protein blots were placed in blocking solution for 2 h and then incubated with Cx43 mouse antibody (1:1000, Abcam, ab79010), p-Cx43 rabbit antibody (1:1000, Abcam, ab30559), GFAP mouse antibody (1:1000, Abcam, ab10062), or glyceraldehyde-3-phosphate dehydrogenase (GAPDH) rabbit antibody (1:2000, Abcam, ab181602) at 4°C overnight. The next day, blots were incubated at room temperature for 1 h with horseradish peroxidase (HRP) secondary antibody (goat anti-mouse or goat anti-rabbit, 1:40000, Santa Cruz, Dallas, TX, USA) and developed by chemiluminescence. Band intensity of the protein sample was obtained by normalizing the density with GAPDH and taken the average of each group. Fifteen mice in total were used in each experimental group with five mice per day for the last 3 days of experiment.

### Statistical Analysis

All data were expressed as mean ± standard error (SE). Statistical differences were analyzed by IBM SPSS statistics software version 18.0 (SPSS Company, Chicago, IL, USA), using one-way analysis of variance (ANOVA) followed by Fisher’s Least Significant Difference (LSD) test or two-way repeated measures ANOVA followed by Bonferroni’s multiple comparison test for more than two groups. Student’s *t* test (2-tailed) was also used between two groups. The criterion for statistical significance was *p* < 0.05 (marked as *).

## Results

### Evaluation of the PWMT and Hind Limb Use Score in Control, Sham, BCP, BCP-Morphine and BTcP Mice

**Figure 1:**
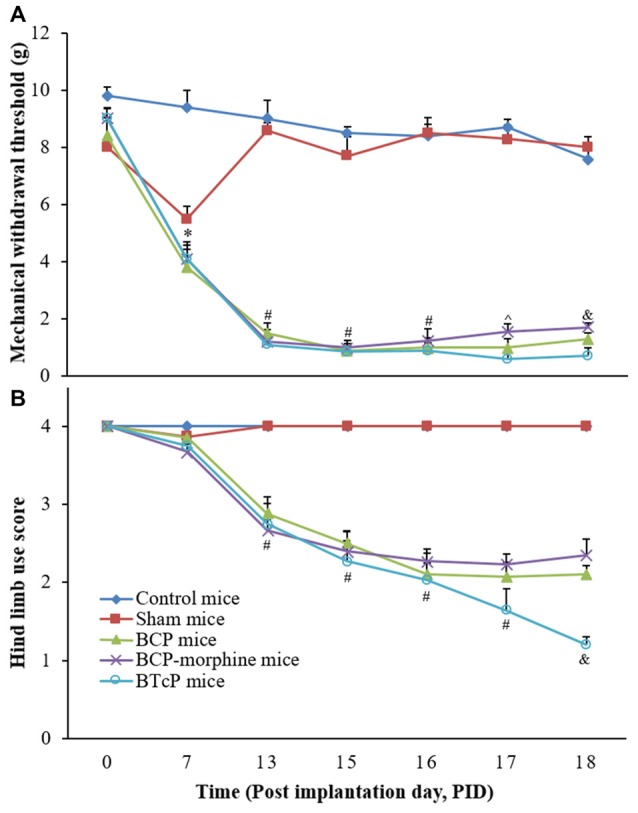
Evaluation of the paw withdrawal mechanical threshold (PWMT) of left hind paw **(A)** and hind limb use score **(B)** in normal, sham, bone cancer pain (BCP), BCP-morphine and Breakthrough cancer pain (BTcP) mice. The tests of PWMT of left hind paw and hind limb use score in mice were performed at 8 am. Five mice were used for each group. *P < 0.05, sham, BCP, BCP-morphine or BTcP mice vs. control mice; ^#^*P* < 0.05, BCP, BCP-morphine, or BTcP mice vs. sham mice; ^∧^*P* < 0.05, BTcP mice vs. BCP-morphine mice; ^&^*P* < 0.05, BTcP mice vs. BCP or BCP-morphine mice.

First, we evaluated the PWMT of the left hind paw and hind limb use score in the control, sham, BCP, BCP-morphine and BTcP mice and the results are shown in Figure 1. There was no significant difference between control mice and sham mice (injected D-Hank’s solution into the femur bone of left hind limb) in the PWMT and hind limb use score except on PID 7 due to puncture operation in the sham mice. However, the PWMT (Figure 1A) and hind limb use score (Figure 1B) were significantly decreased (*p* < 0.05) in BCP, BCP-morphine and BTcP mice compared to that of control and sham mice started on PID 13 and the decreases were persisted from PID 13 through PID 18. Moreover, the PWMT was significantly decreased (*p* < 0.05) in BTcP mice compared to that of BCP-morphine mice in PIDs 17–18 and BCP mice on PID 18 (Figure 1A). The hind limb use score was also significantly decreased (*p* < 0.05) in BTcP mice compared to that of BCP and BCP-morphine mice on PID 18 (Figure 1B). However, despite the presence of decreased PWMT and hind limb use score at the ipsilateral hind limb with LLC tumors, the weights of BTcP mice were similar to that of control mice and without bone fracture during the experimental period.

### The Expressions of GFAP, Cx43 and p-Cx43 in the Spinal Cord in Control, Sham, BCP, BCP-Morphine and BTcP Mice

**Figure 2:**
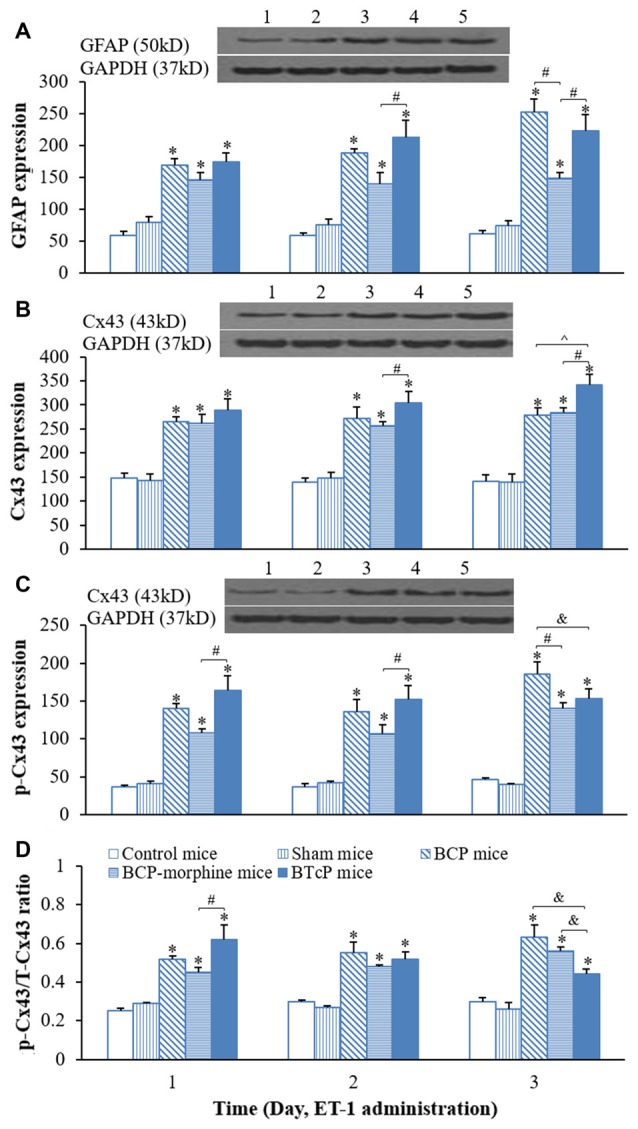
Protein expressions of glial fibrillary acidic protein (GFAP) **(A)** Cx43 **(B)** p-Cx43 **(C)** and the ratio of p-CX43/T-Cx43 **(D)** in normal, sham, BCP, BCP-morphine and BTcP mice. The bands of Western blotting in **(A–C)**: (1) protein extracts from the tissues of spinal cord in a representative control mice; (2) protein extracts from the tissues of spinal cord in a representative sham mice; (3) protein extracts from the tissues of spinal cord in a representative BCP mice; and (4) protein extracts from the tissues of spinal cord in a representative BCP-morphine mice; and (5) protein extracts from the tissues of spinal cord in a representative BTcP mice. Five mice were used for each group. GFAP, Cx43 and p-Cx43 were analyzed by Western blotting and the ratio of p-CX43/T-Cx43 was calculated from phosphonated Cx43 and total Cx43. *P < 0.05, vs. control or sham mice; ^#^*P* < 0.05, vs. BCP-morphine mice; ^∧^*P* < 0.05, vs. BCP mice; ^&^*P* < 0.05, vs. BTcP mice.

After we completed the studies of the PWMT and hind limb use score in control, sham, BCP, BCP-morphine and BTcP mice, next, we investigated the expressions of GFAP, Cx43 and p-Cx43 proteins in the spinal cord in mice and the results are illustrated in Figure 2. The expression of GFAP in the spinal cord was significantly increased (*p* < 0.05) in BCP, BCP-morphine and BTcP mice compared to that of control and sham mice in PIDs 16–18, the amounts of protein were gradually increased over time in BCP and BTcP mice (Figure 2A). Furthermore, the expression of GFAP was significantly higher (*p* < 0.05) in BTcP mice than that of BCP-morphine mice in PIDs 17–18, while the amount of GFAP protein was higher (*p* < 0.05) in BCP mice than that of BCP-morphine mice on PID 18 (Figure 2A).

Similarly, the expression of Cx43 in the spinal cord of mice was also markedly increased (*p* < 0.05) in BCP, BCP-morphine and BTcP mice compared to that of control and sham mice in PIDs 16–18 (Figure 2B). Moreover, the expression of Cx43 protein was significantly higher (*p* < 0.05) in PIDs 17–18 in BTcP mice compared to that of BCP-morphine mice, while the expression was up-regulated without statistical difference (*p* > 0.05) on PID 17 but was significantly higher (*p* < 0.05) on PID 18 in BTcP mice compared to that of BCP mice (Figure 2B). However, there was no significant difference (*p >* 0.05) of Cx43 expression between BCP mice and BCP-morphine mice.

The phosphorylation of Cx43 protein (p-Cx43) was also significantly increased *p* < 0.05 in BCP, BCP-morphine and BTcP mice compared to that of control and sham mice in PIDs 16–18 (Figure 2C). Furthermore, the expression of p-Cx43 protein was significantly increased (*p* < 0.05) in PIDs 16–17 but no statistically significant difference (*p* > 0.05) on PID 18 in BTcP mice compared to that of BCP-morphine mice (Figure 2C). The expression level of p-Cx43 protein was also significantly increased (*p* < 0.05) in BCP mice than that of BCP-morphine and BTcP mice on PID 18 (Figure 2C).

The amount of p-Cx43 is directly related to total amount of Cx43 protein (T-Cx43), therefore, we calculated the ratio of p-Cx43/ T-Cx43. The p/T ratio of Cx43 was significantly increased (*p* < 0.05) in BCP, BCP-morphine and BTcP mice compared to that of control and sham mice in PIDs 16–18 (Figure 2D). The p-Cx43/T-Cx43 ratio was significantly higher (*p* < 0.05) in BTcP mice than that of BCP-morphine mice, but no significant difference (*p* > 0.05) compared to BCP mice on PID 16. However, the p/T ratio was much lower (*p* < 0.05) in BTcP mice than that of BCP and BCP-morphine mice on PID 18 (Figure 2D). There were no significant differences (*p* > 0.05) between control mice and sham mice in the expressions of GFAP, Cx43 and p-Cx43 proteins as well as p/T ratio of Cx43.

### Effects of Gap26 on the PWMT and Hind Limb Use Score in Control, BCP, BCP-Morphine and BTcP mice

Since there was no significant difference between control mice and sham mice in above studies, we excluded sham group from further studies.

**Figure 3:**
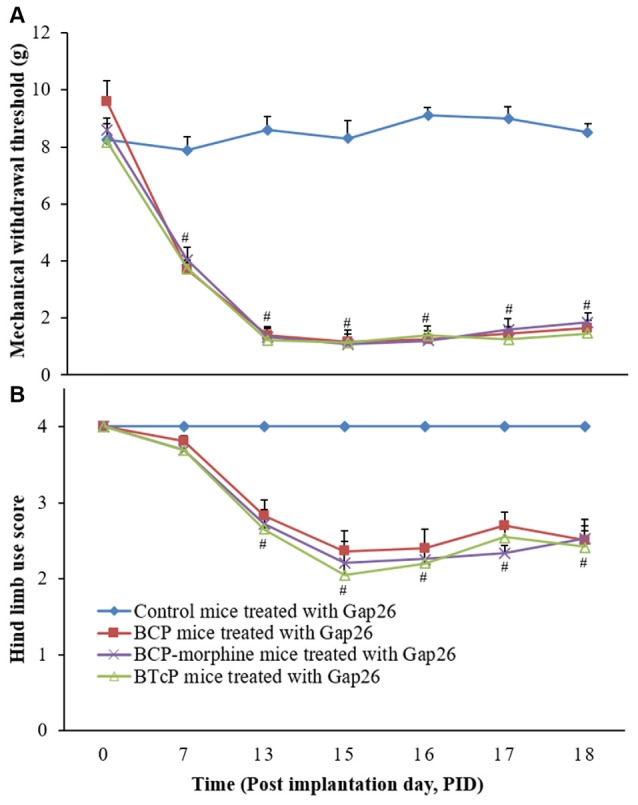
Effects of Gap26 on the PWMT of left hind paw **(A)** and hind limb use score **(B)** in normal, BCP, BCP-morphine and BTcP mice. Gap 26 was administrated by intrathecal injection at 5 mg/kg/day, once a day for 3 days (post-implantation days, PIDs 16–18). The tests of the PWMT of left hind paw and hind limb use score in mice were performed at 8 am. Five mice were used for each group. ^#^*P* < 0.05, BCP, BCP-morphine or BTcP mice vs. control mice.

Gap26 is a Cx mimetic peptide for selectively blocking GJ. Therefore, we investigated the effects of Gap26 (5 mg/kg/day) by intrathecal injection in PIDs 16–18 on the PWMT of the left hind paw and hind limb use score in the control, BCP, BCP-morphine and BTcP mice and the results are shown in Figure 3. The PWMT (Figure 3A) and hind limb use score (Figure 3B) were significantly decreased (*p* < 0.05) in BCP, BCP-morphine and BTcP mice treated with Gap26 compared to that of control mice treated with Gap26 in PIDs 13–18. However, no significant differences (*p* > 0.05) of the PWMT and hind limb use score were observed among the BCP, BCP-morphine and BTcP mice (Figures 3A,B).

**Figure 4:**
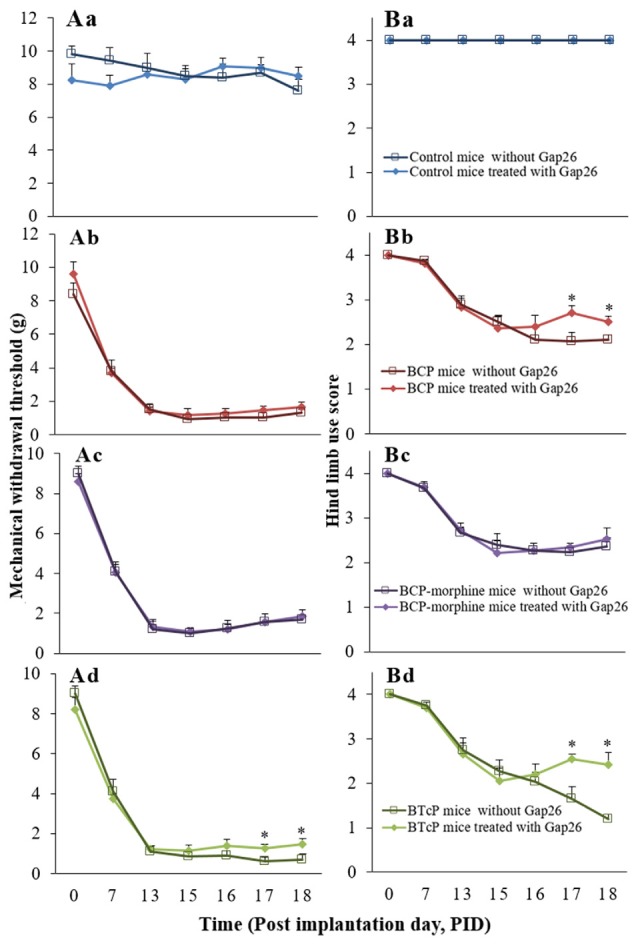
Comparison of the PWMT of left hind paw **(A)** and hind limb use score **(B)** in normal, BCP, BCP-morphine and BTcP mice with or without Gap26 treatment. Gap26 (5 mg/kg/day) was administered by intrathecal injection once a day for 3 days in the last 3 days of experiment (PIDs 16–18). Pain behavioral tests of PWMT of left hind paw and hind limb use score in mice were performed at 8 am. Five mice were used for each group. *P < 0.05, vs. mice without Gap26. The data were combined with the data of Figures 1, 3.

Furthermore, there was no difference in the control mice with or without Gap26 treatment (Figures 4Aa,Ba). The hind limb use score (2.04 ± 0.19 vs. 2.70 ± 0.17 on PID 17; 2.10 ± 0.11 vs. 2.51 ± 0.13 on PID 18) was significantly increased (*p* < 0.05) in BCP mice with Gap 26 treatment in PIDs 17–18 (Figure 4Bb) but no significant difference of the PWMT (Figure 4Ab). There were no statistically significant difference (*p* > 0.05) of the PWMT and hind limb use score in BCP-morphine mice with or without Gap 26 treatment (Figures 4Ac,Bc). However, the PWMT (0.60 ± 0.22 vs. 1.25 ± 0.23 on PID 17; 0.70 ± 0.29 vs. 1.46 ± 0.29 on PID 18) and hind limb use score (1.640 ± 0.28 vs. 2.55 ± 0.11 on PID 17; 1.20 ± 0.11 vs. 2.42 ± 0.28 on PID 18) were markedly increased (*p* < 0.05) in BTcP mice with Gap26 treatment in PIDs 17–18 (Figures 4Ad,Bd).

### Effects of Gap26 on the Expressions of GFAP, Cx43 and p-Cx43 Proteins in the Spinal Cord in Control, BCP, BCP-Morphine and BTcP mice

**Figure 5:**
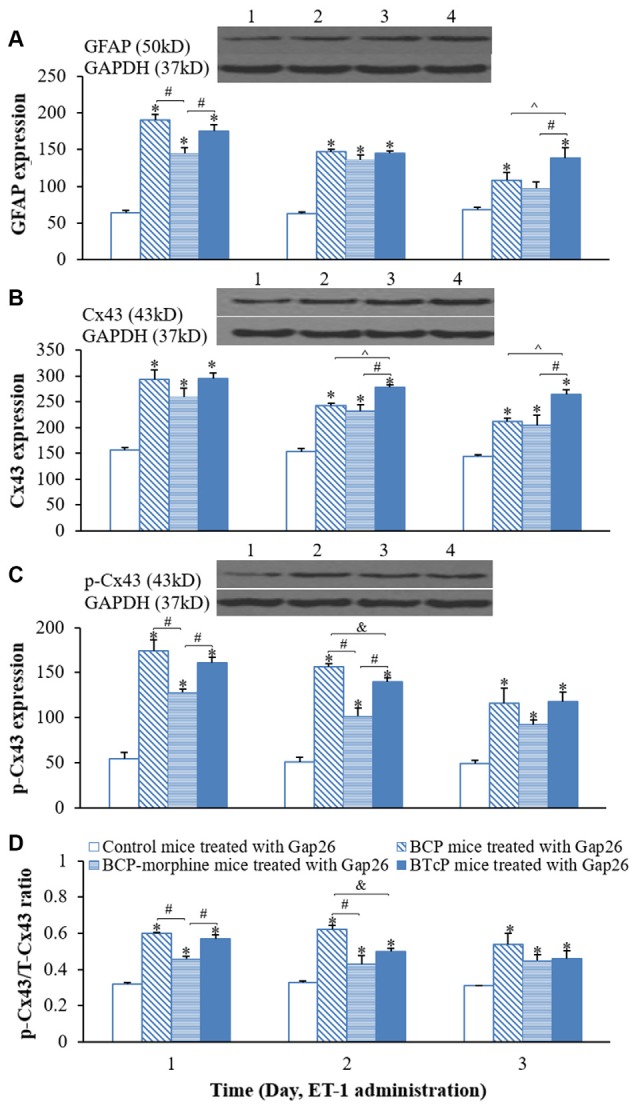
Effects of Gap26 on protein expressions of GFAP **(A)**, Cx43 **(B)**, p-Cx43 **(C)** and the ratio of p-CX43/T-Cx43 **(D)** in normal, BCP, BCP-morphine and BTcP mice. The bands of Western blotting in **(A–C)**: (1) protein extracts from the tissues of spinal cord in a representative control mice treated with Gap26; (2) protein extracts from the tissues of spinal cord in a representative BCP mice treated with Gap26; (3) protein extracts from the tissues of spinal cord in a representative BCP-morphine mice treated with Gap26; and (4) protein extracts from the tissues of spinal cord in a representative BTcP mice treated with Gap26. GFAP, Cx43 and p-Cx43 were analyzed by Western blotting and the ratio of p-CX43/T-Cx43 was calculated from phosphonated Cx43 and total Cx43. Gap 26 was administrated by intrathecal injection at 5 mg/kg/day, once a day for 3 days (PIDs 16–18). Five mice were used for each group. *P < 0.05, vs. control mice; ^#^*P* < 0.05, vs. BCP-morphine mice; ^∧^*P* < 0.05, vs. BCP mice; ^&^*P* < 0.05, vs. BTcP mice.

**Figure 6:**
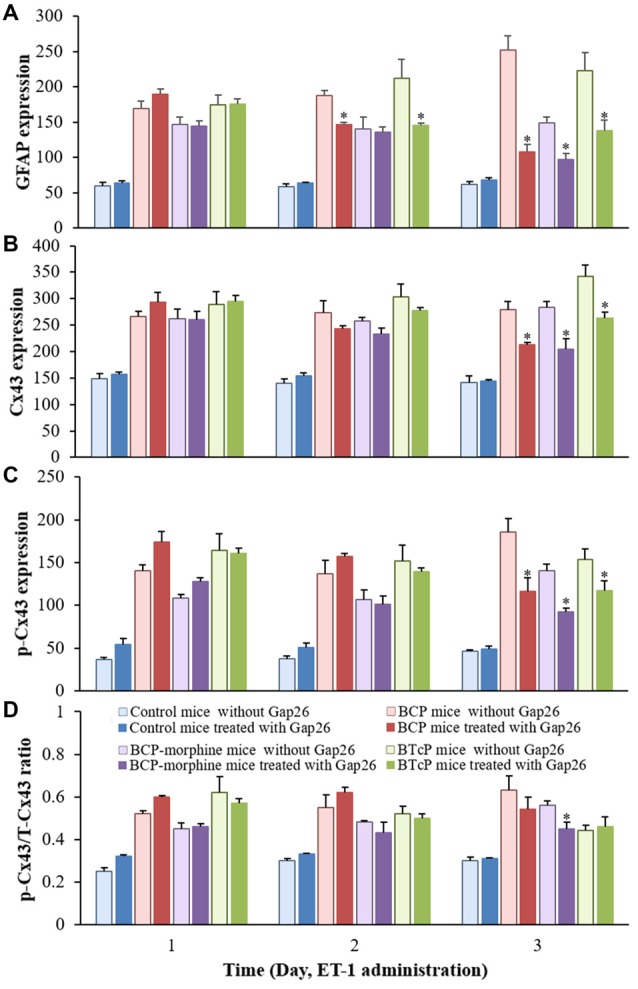
Comparison of the protein expressions of GFAP **(A)**, Cx43 **(B)**, p-Cx43 **(C)** and the ratio of p-CX43/T-Cx43 **(D)** in normal, BCP, BCP-morphine and BTcP mice with or without Gap26 treatment. Gap26 (5 mg/kg/day) was administered by intrathecal injection once a day for 3 days in the last 3 days of experiment (PIDs 16–18). GFAP, Cx43 and p-Cx43 were analyzed by Western blotting and the ratio of p-CX43/T-Cx43p was calculated from phosphonated Cx43 and total Cx43. Five mice were used for each group. *P < 0.05, vs. no Gap26 treatment. The data were combined with the data of Figures 2, 5.

Gap26 peptide maps to Cx43 residue 63–75 to inhibit the activity of Cx43. Therefore, we evaluated the effects of Gap26 on the expressions of GFAP, Cx43 and p-Cx43 in the spinal cord in PIDs 16–18 by Western blotting analysis after Gap26 administration (5 mg/kg/day, daily for 3 days) into the spinal canal and the results are illustrated in Figure 5. The expression of GFAP protein was significantly increased (*p* < 0.05) with Gap26 treatment in BCP and BTcP mice compared to that of control mice (Figure 5A). There was significant higher expression level of GFAP protein (*p* < 0.05) with Gap26 treatment in BCP-morphine mice compared to that of control mice in PIDs 16–17, but GFAP expression was decreased over time with Gap26 treatment and no significant difference (*p* > 0.05) between the two groups on PID 18 (Figure 5A). The expression levels of GFAP protein were significantly decreased (*p* < 0.05) with Gap26 treatment in BCP mice (187.81 ± 15.69 vs. 146.83 ± 6.52 on PID 17; 252.04 ± 45.78 vs. 108.10 ± 23.20 on PID 18) and BTcP mice (212.20 ± 59.43 vs. 145.24 ± 6.64 on PID 17; 222.64 ± 58.26 vs. 138.11 ± 32.22 on PID 18) in PIDs 17–18, and in BCP-morphine mice (148.68 ± 20.20 vs. 96.80 ± 20.51) on PID 18 (Figure 6A).

The expression of Cx43 protein was significantly increased (*p* < 0.05) with Gap26 treatment in BCP, BCP-morphine and BTcP mice compared to that of control mice in PIDs 16–18 (Figure 5B). Furthermore, the expression of Cx43 protein was significantly increased (*p* < 0.05) with Gap26 treatment in BTcP mice compared to that of BCP and BCP-morphine mice in PIDs 17–18 (Figure 5B). However, the expression of Cx43 protein was decreased over time with Gap26 treatment with statistically significant difference (*p* < 0.05) in BCP (279.10 ± 33.16 vs. 212.37 ± 11.74), BCP-morphine (283.44 ± 23.55 vs. 204.51 ± 43.20) and BTcP mice (341.52 ± 50.48 vs. 263.75 ± 23.70) on PID 18 (Figure 6B).

The expression levels of p-Cx43 protein were significantly increased (*p* < 0.05) in BCP, BCP-morphine and BTcP mice compared to that of control mice with Gap26 treatment in PIDs 16–18 (Figure 5C). There was statistically significant difference (*p* < 0.05) among BCP, BCP-morphine and BTcP mice in PIDs 16–17 but no significant difference (*p* > 0.05) on PID 18 with Gap26 treatment (Figure 5C). The expressions of p-Cx43 had no significant difference (*p* > 0.05) in PIDs 16–17 but were significantly decreased (*p* < 0.05) on PID 18 with Gap26 treatment in BCP (185.62 ± 34.91 vs. 115.92 ± 36.72), BCP-morphine (140.20 ± 17.92 vs. 92.25 ± 10.65) and BTcP mice (153.43 ± 27.80 vs. 117.61 ± 23.97) with BCP mice decreased the most (Figure 6C). However, the expressions of GFAP, Cx43 and p-Cx43 proteins have no differences with or without Gap26 treatment in control mice.

The p/T ratio of Cx43 was calculated to evaluate the relative expression level of p-Cx43. The ratio of p-Cx43/T-Cx43 was significantly increased (*p* < 0.05) in BCP, BCP-morphine and BTcP mice compared to that of control mice with Gap26 treatment in PIDs 16–18. There was significant difference (*p* < 0.05) among BCP, BCP-morphine and BTcP mice in PIDs 16–17 but no significant difference (*p* > 0.05) on PID 18 (Figure 5D). Furthermore, the p/T ratio of Cx43 was significantly reduced by Gap26 treatment in BCP-morphine mice (0.56 ± 0.05 vs. 0.45 ± 0.07) on PID 18 (Figure 6D).

## Discussion

The association for Palliative Medicine (APM) of Great Britain and Ireland task group has defined BTcP as “a transient exacerbation of pain that occurs either spontaneously, or in relation to a specific predictable or unpredictable trigger, despite relatively stable and adequately controlled background pain” (Davies et al., 2009). We previously established a reliable and reproducible mouse model of BTcP with metastatic BCP (Tang et al., 2016). We employed this model to study the role of the expression and phosphorylation of Cx43 protein in lumbar spinal cord in BTcP mice and have demonstrated that Cx43 played an important role in the pathogenesis of BTcP for the first time. In the establishment of BTcP model, we found that local injection of ET-1 at 18 μg/kg in the thigh of normal mice did not cause BTcP-like behaviors; however, local injection of the same concentration of ET-1 in the thigh of LLC tumor bearing mice even with good analgesic effect of morphine induced intense pain behaviors with dense production and synchronization of action potentials recorded by inserted electrodes in different segments of the spinal dorsal horn (data not shown). These results suggest that the production of abnormal pain in BTcP mice seems not only to be simply associated with long-term potentiation (LTP) of spinal dorsal horn in the central sensitization of chronic pain, but also related to inflammatory and neuropathic pain and other factors (Berker and Dinçer, 2005).

In the present studies, we observed that the decreases of the PWMT and hind limb use score in LLC bearing mice without treatment (BCP mice), treated with morphine (BCP-morphine mice), or morphine + ET-1 (BTcP mice) were associated with the infiltration of lung cancer cells in the hind paw of mice by immunohistochemical analysis (data not shown), suggesting the mouse model of metastatic BCP was established. The significant reduction in the two pain behavioral tests of the PWMT and hind limb use score in BTcP mice with enhanced electrophysiology of the ipsilateral sciatic nerve within 30 min after ET-1 injection showed that the BTcP model was successfully established (Tang et al., 2016). However, the pain behavioral indexes were not statistically different until on the third day of ET-1 injection (PID 18) in BCP mice, BCP-morphine mice and BTcP mice, the possible mechanism may be due to the delay of the production of functional proteins or regulation of relevant channel function in the nervous system of BTcP mice induced by ET-1.

Astrocytes are specialized glial cells and the most abundant cells in the central nervous system, the cells play an important role for maintaining the microenvironment of nervous system by regulating energy metabolism, synaptic development, neurotransmitter uptake and neuronal signaling (Verkhratsky et al., 2016). The spinal cord is the first relay station that receives input of sensory fiber information from the nociceptor. The integration of nociceptive and non-nociceptive information occurs in the Rexed laminae of the spinal dorsal horn which plays an important role in the plasticity of sensory information processing and transformation (Pan and Pan, 2004).

Numerous studies have shown that activation of astrocytes plays an important role in different types of pain due to various causes such as drugs (Liu et al., 2016), machinery (Gao et al., 2016), inflammation (Fang-Hu et al., 2015), virus (Shi et al., 2012) and other related pathogenies. GFAP is a major component of the cytoskeletal intermediate filament and is highly expressed in mature or activated astrocytes of the central nervous system (Middeldorp and Hol, 2011). Thus, GFAP acts as an important sign of astrocyte-activation. Previous studies have shown that the activation of microglia and astrocytes in the spinal cord played an important role in the maintenance of allodynia in cancer pain (Wang X.-W. et al., 2012; Lu et al., 2015). It was reported that the overexpression of GFAP in astrocytes in spinal dorsal horn was associated with neuropathic pain and it may not be related to BCP (Ducourneau et al., 2014). Interestingly, in the present study, we observed that GFAP expression in spinal cord was down-regulated in BCP mice with morphine treatment. However, a study of morphine tolerance (Shen et al., 2014) has shown that chronic activation of astrocytes for more than 7 days in the spinal dorsal horn was observed after morphine treatment in an animal model, which is in contrast to the present study. The possible reason may be due to different experimental design. Therefore, we hypothesize that activation of astrocytes in spinal cord was inhibited by morphine but may be chronically activated with prolonged morphine presence, leading to morphine tolerance to induce pain. In the present study, we also observed that the expression of GFAP was significantly higher in BTcP mice than that of BCP-morphine mice, indicating that stronger activation of astrocytes in BTcP mice was less likely to be inhibited by morphine. The specific mechanism involved in the pain process needs to be further investigated.

An important feature of spinal dorsal horn is the high expression of Cx in signal transduction and substance exchange, which is the basic functional protein of GJ channel. Under pathological conditions, there are homotypic and heterotypic GJs that are formed by homotypic or heterotypic Cx between astrocytes or astrocytes and neurons, then, the cells constitute a more extensive functional aggregation, so astrocytes can have rapid electrical response to the activation of neurons with highly plasticity and bi-directionality (Chever et al., 2016).

Studies have suggested that Cx43 played an important role in different types of pain. The expression of Cx43 in glial cells was significantly increased and contributed to allodynia after dorsal root ganglion nerve injury in neuropathic pain or low-back pain (Zhang et al., 2009; Warwick and Hanani, 2013). Moreover, inhibition of Cx43 expression in the trigeminal ganglion could reduce trigeminal pain (Ohara et al., 2008). In the present study, the expression of Cx43 protein was up-regulated in BTcP mice. Gap26 could reduce the occurrence of BTcP evidenced by the improvements of the PWMT and hind limb use score via down-regulating the expression of Cx43 protein, suggesting that the overexpression of Cx43 protein in spinal cord may play an important role in the development of BTcP. As an important part of GJ, Cx43 also participates in the material, energy and ion exchange (Alexander and Goldberg, 2003). The function of Cx43 channel is affected by multiple factors, such as intracellular pH value, Ca^2+^ concentration, Cx phosphorylation state, cross-channel voltage and some neurohumoral factors. Studies have shown that regulation of Cx43 channels exhibited a biphasic-dependence on intracellular calcium concentration, when the concentrations were below 500 nM to cause their activation, while the concentrations were above 500 nM to trigger negative feedback loops (De Bock et al., 2012; Wang N. et al., 2012). In addition, when the intracellular Ca^2+^ concentration and pH value decreased simultaneously, the conductivity of GJ channels was further decreased (Sáez et al., 1997). Rapid regulation of the channel function of Cx43 protein plays a role in short-term and paroxysmal BTcP. However, some studies have also shown that the addition of activated microglia or mixed cytokines down-regulated the expression of Cx43 in cultured astrocytes but increased the probability of hemichannel opening (Même et al., 2006). There are two channel forms of Cx43 protein including GJ and hemichannel, suggesting that different functional forms of Cx43 protein may have different regulatory mechanisms.

Notably, the activation of astrocytes indicated by overexpressions of GFAP and Cx43 was not necessarily synchronized. Xu et al. (2014) observed that increased GFAP level and decreased expression of Cx43 in astrocytes of spinal cord in spinal nerve ligation (SNL) rats. Therefore, different causes and types of nerve injury may differently regulate the expression of Cx43 in spinal cord.

It has been reported that a variety of protein kinases such as PKA, PKC, PKG, MAPK, CK1, and others could phosphorylate the related sites of the Cx43 protein directly or indirectly, thus affecting the GJ function (Rackauskas et al., 2010). In the present study, we have showed that the high expression of Cx43 protein with different phosphorylation levels in BCP mice, BCP-morphine mice and BTcP mice, therefore the p/T ratio of Cx43 protein was more noteworthy. We found that the expressions of Cx43 and p-Cx43 proteins were higher at early stage but the p/T ratio of Cx43 was decreased gradually over time in BTcP mice. Study has showed that increased Cx43 phosphorylation at the site of serine 368 was associated with the functional down-regulation of GJ (Palatinus and Gourdie, 2016). In the present study, the decreased p/T ratio of Cx43 could lead to the occurrence of BTcP by regulating the function of GJ channels, and the p/T ratio of Cx43 was an important factor affecting GJ function.

It has been proved that GJ channel function was affected by Cx43 phosphorylation (Quesseveur et al., 2015). Study has shown that cyclic guanosine monophosphate phosphorylated Ser257 of Cx43 reduced intracellular conduction and dye permeability in cardiomyocytes of rat (Giepmans, 2004). Epidermal growth factor (EGF; Cottrell et al., 2003) or high glucose (Fernandes et al., 2004) could phosphorylate the main sites of Cx43 such as S255, S279, S282 to reduce channel opening probability and inhibit GJ function. Our results are consistent with the report (Figures 2, 5, 6). However, not all Cx43 phosphorylation can down-regulate GJ channel function. Yogo et al. (2006) found that hyperphosphorylation status of multiple sites (Ser365, Ser368, Ser369, Ser373, etc.) of Cx43 induced by follicle-stimulating hormone could enhance channel formation and function in granulosa cells of rat. The possible cause may be due to that Cx43 phosphorylation in different cells may regulate channel function differently. However, the exact mechanisms remain to be further investigated.

In the present studies, we have shown that the expressions of Cx43 and p-Cx43 were significantly down-regulated (*p* < 0.05) but the p/T ratio of Cx43 was not statistically significant in difference (*p* > 0.05) by Gap26 treatment in BTcP mice accompanied by the improvement of the PWMT and hind limb use score (Figures 3–6). These results suggest that inhibition of the expression of Cx43 protein by Gap26 could only partly control the occurrence of BTcP. The possible explanation is that Gap26 can map to the amino acid residue 263–275 of Cx43 protein to suppress Cx43 function for further decrease of the number of functional GJ channels. The carboxyl terminal of Cx43 protein is the main region of phosphorylation located at 241–382 sites. The phosphorylation levels of amino acid residues such as serine, threonine and tyrosine determine the permeability and function of GJ channel, so Gap26 also has an impact on the expressions of Cx43 and p-Cx43 by acting on the phosphorylation sites of Cx43. Thus, the total amount of Cx43 protein and its phosphorylation level play a role in regulating the formation and function of GJ channels.

The levels of Cx43 and p-Cx43 proteins were significantly decreased (*p* < 0.05) but the p/T ratio had no significant change by Gap26 treatment with increased hind limb use score in BCP mice (Figures 3–6), suggesting that the high expression of Cx43 protein but not the phosphorylation of Cx43 affected the course of BCP and inhibition of the expression of Cx43 by Gap26 improved the pain tolerance. The result is consistent with previous report by Hang et al. (2016) which showed that Gap26 could significantly attenuate mechanical allodynia in rats with BCP. The possible reason may be due to different presentations of pathogenesis in BCP and BTcP. In addition, the expression of GFAP was also significantly down-regulated (*p* < 0.05) by Gap26 in our study (Figures 5, 6). The result is consistent with the studies by Roh et al. (2010), that down-regulation of GJ function by Cx43 was associated with decreased activation of astrocytes in spinal dorsal horn to suppress mechanical allodynia and thermal hyperalgesia. One of the limitations in the present studies is that we did not use astrocytes but used homogenized tissues of spinal cord of mice. We will use astrocytes for our future studies of the possible mechanisms associated with BTcP.

## Conclusion

The present studies have demonstrated that the expression and phosphorylation of Cx43 protein in the spinal cord are involved in the development and maintenance of BTcP via regulating GJ function in mice for the first time. Phosphorylation of certain Cx sites is sufficient to alter the function and important protein sites of GJ channels and plays an important role in pathogenesis of BTcP by regulating the communication of GJ. Our studies have significant impact on the study of the possible mechanism of BTcP and may provide scientific rationale and a novel approach for the treatment of BTcP clinically. However, the process of BTcP is complex and involved in multiple mechanisms; it needs to be further investigated.

## Author Contributions

DH and XY designed and supervised the studies. XL and SJ performed cell culture and Western blotting. XL and QL performed animal experiments. XL and HY analyzed the data. XL, SJ and SC wrote the manuscript. All authors read and approved the final manuscript.

## Conflict of Interest Statement

The authors declare that the research was conducted in the absence of any commercial or financial relationships that could be construed as a potential conflict of interest.

## Abbreviations

ANOVA: one-way analysis of variance
APM: association for palliative medicine
BCP: bone cancer pain
BTcP: breakthrough cancer pain
Cx: connexin
DD Water: distilled deionized water
EGF: epidermal growth factor
ET-1: endothelin-1
FBS: fetal bovine serum
GAPDH: glyceraldehyde-3-phosphate dehydrogenase
GFAP: glial fibrillary acidic protein
GJ: gap junction
HRP: horseradish peroxidase
i.p.: intraperitoneal
LLC: Lewis lung cancer
LSD: least significant difference
LTP: long-term potentiation
NS: normal saline
NRS: numeric rating scale
PID: post-implantation day
PK: protein kinase
PWMT: paw withdrawal mechanical threshold
SE: standard error
SNL: spinal nerve ligation
SPF: specific-pathogen-free
VS: versus.
